# Reliability and Reproducibility of Metabolite Quantification Using ^1^H MRS in the Human Brain at 3 T and 7 T

**DOI:** 10.1002/nbm.70087

**Published:** 2025-07-08

**Authors:** Zeinab Eftekhari, Thomas B. Shaw, Dinesh K. Deelchand, Małgorzata Marjańska, Wolfgang Bogner, Markus Barth

**Affiliations:** ^1^ ARC Training Centre for Innovation in Biomedical Imaging Technology (CIBIT) The University of Queensland Brisbane Queensland Australia; ^2^ Centre for Advanced Imaging The University of Queensland Brisbane Queensland Australia; ^3^ School of Electrical Engineering and Computer Science The University of Queensland Brisbane Queensland Australia; ^4^ Centre for Magnetic Resonance Research, Department of Radiology University of Minnesota Minneapolis Minnesota USA; ^5^ Christian Doppler Laboratory for MR Imaging Biomarkers (BIOMAK), Department of Biomedical Imaging and Image‐Guided Therapy Medical University of Vienna Vienna Austria; ^6^ High Field MR Centre (HFMR), Department of Biomedical Imaging and Image‐guided Therapy Medical University of Vienna Vienna Austria; ^7^ Comprehensive Centre for AI in Medicine (CAIM) Medical University of Vienna Austria

**Keywords:** CV, ICC, longitudinal, MND, MRS, sLASER, STEAM, ultra‐high field

## Abstract

Proton magnetic resonance spectroscopy (^1^H MRS) is a valuable non‐invasive technique for quantifying biochemical compounds in vivo, facilitating the monitoring of disease progression and treatment. This study evaluates the reliability and reproducibility of ^1^H MRS measurements by comparing data acquired with STEAM and sLASER sequences at 3 T and 7 T. We assessed test–retest reliability and reproducibility by scanning healthy participants twice with STEAM and sLASER at 3 T and 7 T in two different voxel locations about 1 week apart, investigating the impact of measurement conditions on results. Reliability was measured using the intraclass correlation coefficients (ICC), whereas reproducibility was assessed with the coefficients of variation (CV). Our findings reveal that data acquired with the sLASER sequence show superior reliability and reproducibility compared to STEAM for most metabolites at both fields. Although the higher field strength of 7 T provides advantages in signal‐to‐noise ratio and resolution as expected, our study highlights that a field strength of 3 T provides a suitable alternative when ultra‐high‐field scanners are unavailable. This study provides valuable insights for researchers regarding the selection of appropriate MRS sequences and field strengths based on reliability and reproducibility. Our findings underscore the importance of consistent measurements over time, guiding decisions in longitudinal studies and enhancing data consistency.

## Graphical abstract

This study's findings offer valuable insights for researchers conducting longitudinal studies using MRS. The improved reliability and reproducibility of the sLASER technique, particularly at 7 T, enable more precise tracking of disease progression, potentially leading to improved disease tracking.

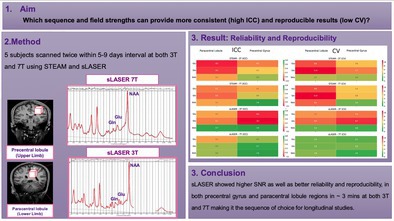

**Abbreviations**
AFP: adiabatic full‐passage
Cho: choline
CP: Carr–Purcell
Cr: creatine
CRLB: Cramér–Rao lower bound
CSDE: chemical shift displacement error
CSF: cerebrospinal fluid
CV: coefficient of variation
FWHM: full width at half maximum
GABA: γ‐aminobutyric acid
Gln: glutamine
Glu: glutamate
Glx: Glu + Gln
GM: grey matter
GOIA: gradient offset independent adiabatic
GPC: glycerophosphocholine
GSH: glutathione
ICC: intraclass correlation coefficient
Lac: lactate
MND: motor neuron disease
myo‐Ins: *myo*‐inositol
NAA: *N*‐acetylaspartate
NAAG: *N*‐acetylaspartylglutamate
OVS: outer volume suppression
PCho: ghosphorylcholine
PCr: phosphocreatine
PE: phosphoethanolamine
ppm: parts per million
PRESS: point resolved spectroscopy
SAR: specific absorption rate
sLASER: semi‐localization by adiabatic selective refocusing
SNR: signal‐to‐noise ratio
STEAM: stimulated echo acquisition mode
Tau: taurine
tCho: total choline (GPC + PCho)
tCr: total creatine (Cr + PCr)
TE: echo time
tNAA: total NAA (NAA + NAAG)
TR: repetition time
UHF: ultra‐high field
VAPOR: variable power RF pulses with optimized relaxation delays
WM: white matter

## 1 Introduction

Proton MR spectroscopy (^1^H MRS) is a non‐invasive technique to quantify biochemical compounds in humans in vivo. As an MR‐based method, it avoids ionizing radiation, making it a suitable method to assess changes in metabolite concentrations, such as monitoring disease progression and treatment [1, 2], or longitudinal studies in healthy participants [1, 2, 3]. Interpreting longitudinal metabolite concentrations requires a clear understanding of the sources of measurement variability. The coefficient of variation (CV, %) captures reproducibility, reflecting the stability of metabolite concentrations across different conditions, set‐ups and sessions [4], which is vital when monitoring metabolite changes within individuals over time. In contrast, the intraclass correlation coefficient (ICC) captures reliability, indicating how well‐repeated measurements distinguish between individuals despite variability [3], which is particularly important in group comparisons or when establishing biomarkers. Together, these metrics allow researchers and clinicians to determine whether observed metabolite concentration changes are due to true biological effects or the influence of the acquisition method (“MRS sequence”) and its parameters, voxel location, and magnetic field strength [1, 3, 5, 6].

Having been developed over the last decades, Stimulated Echo Acquisition Mode (STEAM) and semi‐localization by adiabatic selective refocusing (sLASER) sequences remain widely used as the workhorses for MRS research and clinical applications [7, 8]. There is still an ongoing debate about which sequence is better suited for different research purposes, and this study aims to provide additional evidence for aspects of reliability and reproducibility at different field strengths. Few studies have employed ICC for reliability [6, 9, 10, 11], and the CV for reproducibility, with most focusing on one field strength or MR sequence [5, 6, 9, 10, 12, 13, 14, 15, 16, 17, 18, 19]. A recent study focused on repeatability between these sequences at 7 T by reporting CV with measurements taken within the same session [20]. In contrast, our study investigated the reliability and reproducibility using two acquisition methods (STEAM and sLASER sequences) at 3 T and 7 T by scanning a group of healthy participants twice, approximately 1 week apart, using the same scanners and sequences, better reflecting real‐world longitudinal studies. Two brain regions—the precentral gyrus and the paracentral lobule—were selected, as these areas will be the focus of our future research investigating metabolic changes in motor neuron disease (MND), reflecting the affected regions in certain subgroups of MND.

3 T MRI scanners are widely available, making them suitable for straightforward clinical applications if reliability is sufficient [21, 22]. In contrast, ultra‐high‐field (UHF) scanners (7 T and above) have gained popularity among researchers due to their advantages, primarily higher signal‐to‐noise ratio (SNR), which enables shorter acquisition times and improved spectral and spatial resolution [23]. However, increased B_0_ introduces technical challenges, such as more inhomogeneous transmit (B_1_) fields, larger spatial chemical shift displacement error (CSDE), and higher radiofrequency power deposition (specific absorption rate [SAR]) [24, 25, 26].

Advanced MRS techniques like sLASER [27] are less sensitive to B_1_ inhomogeneity and enhance SNR at the expense of higher SAR compared to point resolved spectroscopy (PRESS) or STEAM [27, 28, 29] sequences. STEAM provides a shorter echo time (TE) than sLASER [30, 31], minimizing signal loss due to T_2_ relaxation and *J*‐evolution, but has an inherent 50% signal loss compared to spin‐echo sequences [29, 32].

The overarching aim of this study was to assess the reliability and reproducibility for quantification of major metabolites using both STEAM and sLASER at 3 T and 7 T within two different locations in the human motor cortex in the right hemisphere, that is, the precentral gyrus and the paracentral lobule. By providing both these metrics, we aim to present a more detailed assessment of sequence performance and enhance the statistical rigor of their assessment.

## 2 Materials and Methods

### 2.1 Hardware

This study was performed on a 3 T Prisma^Fit^ MR scanner with a 64‐channel receive‐only head and body transmit coil (Siemens Healthineers, Erlangen, Germany) as well as on a 7‐T whole‐body MR scanner (MAGNETOM 7 T Plus, Siemens Healthineers, Erlangen, Germany) and 1‐transmit 32‐receive head coil (Nova Medical, Wilmington, MA, USA). It should be noted that the 64‐channel head coil used at 3 T is not standard and is expected to provide higher SNR compared to more commonly used coils with reduced number of receive elements (e.g., 24 or 32).

### 2.2 Phantom Experiments

To optimize parameters, establish protocols and assess system stability, we quantified brain metabolites using a brain‐mimicking uniform aqueous phantom, namely, SPECTRE (Gold Standard Phantoms, London, UK). The SPECTRE phantom includes seven brain metabolites with known concentrations in mmol (glutamate [Glu], *N*‐acetylaspartate [NAA], γ‐aminobutyric acid [GABA], creatine [Cr], lactate [Lac], choline [Cho], *myo*‐inositol [myo‐Ins]) at physiological concentrations and pH. The phantom was scanned in five sessions on different days using two short TE sequences with parameters provided in section 2.4. Voxels (2.5 × 2.5 × 2.5 cm^3^) were consistently positioned at A‐P 20, R‐L 20, and F‐H 20 off iso‐centre. Post‐processing methods are identical to those employed in the human study described in Sections 2.5, 2.7.

### 2.3 Human Subjects

Five healthy subjects (25–33 [m = 29, SD = 2.8] years, three females) were scanned twice at both 3 T and 7 T, with 5–9 days between measurements.

### 2.4 Data Acquisition and Sequence Parameters

**FIGURE 1:**
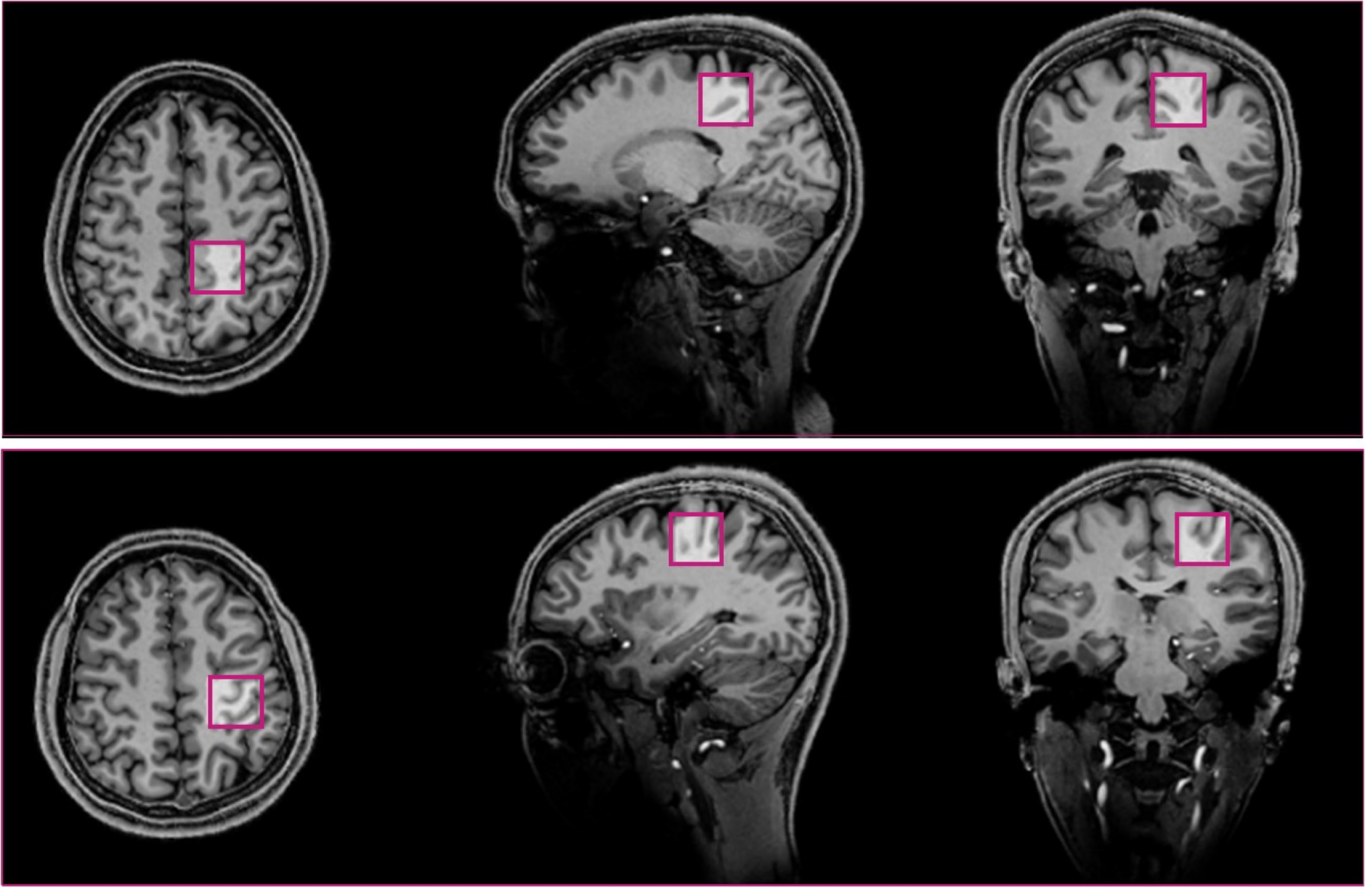
Volumes of interest (VOIs) positioned in the paracentral lobule (top) and the precentral gyrus (bottom) shown on T_1_‐weighted images acquired at 7 T.

Participants underwent an anatomical T_1_‐weighted MP2RAGE scan for voxel positioning and tissue segmentation [33] with the following parameters: at 7 T, voxel size = 0.8 × 0.8 × 0.8 mm^3^, TR = 4300 ms, TE = 2.4 ms, TA = 6 min, FA = 5°, matrix size = 192 × 224 × 256; at 3 T, voxel size = 0.9 × 0.9 × 0.9 mm^3^, TR = 1900 ms, TE = 2.3 ms, TA = 4 min, FA = 9°, matrix size = 176 × 240 × 256 [33]. Before the MRS acquisitions, B_0_ field homogeneity was optimized by performing B_0_ shimming for first‐ and second‐order terms using FAST (EST)MAP at the voxel location of interest [34, 35] across all subjects and sessions consistently with one linear iteration, one all shim iteration and one additional linear iteration. At both fields, each shimming procedure was repeated twice to ensure convergence and stability of the B_0_ field. In addition, the optimization of the RF transmitter power and the water‐suppression variable power RF pulses with optimized relaxation delays (VAPOR) flip angle were completed for each VOI and both sequences. RF transmitter power was optimized by incrementally increasing power while monitoring the water signal intensity, with the system automatically selecting the setting that yielded the maximum signal [27]. Similarly, VAPOR was calibrated by adjusting the flip angle to minimize the residual water signal, with the optimal flip angle selected automatically accordingly. Moreover, VAPOR was interleaved with outer volume suppression (OVS) pulses to suppress unwanted coherences [36]. All ^1^H MRS spectra were acquired in the same order for all sessions at both fields using the parameters in Table 1 for both in vivo and phantom experiments [27, 30, 37]. For phase cycling, STEAM used Siemens' 16‐step EXORCYCLE scheme. For sLASER, the phase cycling scheme can be found in Table S1 of Deelchand et al. [27]. The study aimed to use optimized parameters at each field strength for sufficient SNR within a practical timeframe, aligning the total time across fields. This led to a varying number of transients. A non‐suppressed water spectrum with the same parameters was acquired for eddy current correction and internal water referencing (VAPOR and OVS schemes turned off). Voxels were positioned within the motor cortex in the right hemisphere (i.e., precentral gyrus [M1]; upper limb motor homunculus region and paracentral lobule; lower limb motor homunculus region; see Figure 1). To minimize operator variability, technologists followed strict voxel placement guidelines and printouts of the voxel positioning from the first visit were provided to ensure consistency. The same settings and parameters were applied for the second MRS sessions. Siemens raw data was saved for further post‐processing offline.

**TABLE 1:** Parameters for STEAM and sLASER sequences.

Sequence	Field strength	Voxel size (cm^3^)	TR (s)	TE (ms)	TM (ms)	90° RF bandwidth (Hz)	AFP RF bandwidth (Hz)	Transients	TA (min)
STEAM	3 T	2.5 × 2.5 × 2.5	2	10	43	2500	N/A	64	2.5
	7 T	2.5 × 2.5 × 2.5	8	8	32	6000	N/A	16	2.5
sLASER	3 T	2.5 × 2.5 × 2.5	2	28	N/A	4000	45,000	64	2.5
	7 T	2.5 × 2.5 × 2.5	8	26	N/A	6000	45,000	16	2.5

### 2.5 Data Processing

The MRSinMRS checklist can be found in Table S7. All post‐processing steps were automated using Osprey (v. 2.5.0), an open‐source MRS analysis toolbox, based on the MATLAB (MATLAB R2022a, MathWorks, Natick, MA, USA) platform with the integrated LCModel (linear‐combination model) fitting algorithm [38, 39]. Osprey processing pipeline for single‐shot spectra comprised the following steps: (1) coil combination of raw data, (2) single‐shot correction for frequency and phase changes, (3) eddy‐current correction using the water reference scan, (4) averaging, (5) elimination of residual water and (6) baseline correction [38]. Tissue segmentation (grey matter [GM], white matter [WM] and cerebrospinal fluid [CSF]) was conducted using Statistical Parametric Mapping's toolbox for MATLAB (SPM12 [40]) invoked by Osprey. Data quality metrics including SNR of total creatine (tCr: Cr + PCr) and linewidth of water from the post‐processed summed metabolite spectra were extracted from Osprey. SNR was determined by dividing the metabolite peak height of tCr by the standard deviation of noise within the range of −2 to 0 parts per million (ppm), as peak height provides a more robust measure less influenced by factors such as line broadening and baseline definition compared to peak integrals [4]. Linewidth was defined as the full width at half maximum (FWHM) of a Lorentzian peak model for the water peak between 4.4 and 5.0 ppm.

### 2.6 Spectral Fitting and Quantification

The post‐processed summed metabolite spectra data were fitted using LCModel, a widely used tool for estimating metabolite signal intensities, which is embedded in the Osprey [39]. Spectra were analysed within the chemical shift range of 0.5 to 4.2 ppm [29], employing a knot spacing (DKNTMN) of five for a flat spline baseline and NISMUL = 0 to prevent LCModel from simulating macromolecular components [41]. For 3 T spectra, a gap from 1.1 to 1.85 ppm was used to account for potential lipid contamination. The LCModel basis sets for 3 T and 7 T included 19 simulated metabolites and a measured macromolecule spectrum as previously reported [5, 42, 43]. A recent study showed the feasibility of using a general macromolecule spectrum to fit spectra from different brain regions [44, 45]. Subsequently, Osprey software was employed for metabolite quantification, which used LCModel outputs, unsuppressed water and tCr signals as internal concentration references. The reported metabolite ratios to tCr were determined without applying additional relaxation and tissue composition corrections. However, it is important to note that corrections for tissue fraction (GM, WM and CSF), T_1_ and T_2_ relaxation times for both water and metabolites were incorporated into the calculations of metabolite molal concentrations, following the methodology proposed by Gasparovic [38, 46, 47].

### 2.7 Statistical Analysis

The mean ± SD of Cramér–Rao lower bounds (CRLBs) and metabolite concentrations were calculated using both sessions (*n* = 10). Metabolites with CRLBs higher than 50% were excluded to ensure reliable and accurate quantification in line with previous literature to avoid overly aggressive rejection of low‐concentration metabolites [48]. Metabolites that consistently exhibited a strong negative correlation coefficient (*r* < −0.5) were reported as combined sums: Cr and phosphocreatine (PCr) and phosphocholine (PCho) and glycerophosphocholine (GPC) at 7 T and Cr and PCr, PCho and GPC and NAA and *N*‐acetylaspartylglutamate (NAAG) at 3 T.

To access reliability, we utilized a two‐way random model where each measurement, $x_{\mathrm{ij}}$, for the ith subject in the jth session is expressed as [49]

$$x_{\mathrm{ij}}=\mu +r_{i}+c_{i}+v_{\mathrm{ij}}$$

where μ is the overall mean, $\mu +r_{i}$ is true value for subject i, $c_{j}$ describes a bias error, common to all measurements in session j and $v_{\mathrm{ij}}$ represents the random error. These components are assumed to be sampled from independent normal distributions with variance $\sigma_{r}^{2}$, $\sigma_{c}^{2}$ and $\sigma_{v}^{2}$, respectively.

ICC is defined as the ratio of the variance of interest to the sum of the variance of interest and error variance. In an ideal scenario, we expect all the variance in the experimental data come from between‐subject differences, not from error terms, so $x_{\mathrm{ij}}$ would naturally vary between subjects. Therefore, when the number of subjects is very large, the model 2 ICC, denoted by $\rho_{2A}$, is given by [49]

$$\rho_{2A}=\frac{\sigma_{r}^{2}}{\sigma_{r}^{2}+\sigma_{c}^{2}+\sigma_{v}^{2}}$$

We need to estimate the unknown values of $\sigma_{r}^{2}$, $\sigma_{c}^{2}$ and $\sigma_{v}^{2}$ from a limited sample of subjects randomly selected from the population (in our experiment, we selected five subjects). The estimation of $\rho_{2A}$, denoted by $\mathrm{ICC}\left(A,1\right)$, can be obtained as [49]

$$\rho_{2A}\approx \mathrm{ICC}\left(A,1\right)=\frac{\text{MSBS}-\text{MSWS}}{\text{MSBS}+\left(k-1\right)\times \text{MSWS}}$$

where k, MSBS and MSWS are number of measurements, the mean square between subjects and mean square within subjects. The details derivation of MSBS and MSWS can be found in appendix 1 of [49]. This estimation improves with a larger sample size, but with only five subjects, there is a chance that MSBS is smaller than MSWS and as a result $\mathrm{ICC}\left(A,1\right)$ would be negative [50, 51]. We utilized the R package ‘irr’ to calculate ICC [50, 51]. An ICC close to 1 indicates minimal error and excellent measurement reliability, suggesting that variability primarily arises between subjects rather than within sessions. Conversely, a lower value indicates a higher measurement error and is categorized as per the following: 0.9–0.7 excellent; 0.7–0.6 good; 0.6–0.4 fair; < 0.4 poor [52, 53, 54].

CVs were calculated in line with previous work (Wijtenburg et al. 2018) to assess reproducibility for both metabolite ratios to tCr and concentration estimates for each sequence at different field strengths [12, 55]. The individual CV (in %) was computed by first calculating the CV for each participant as the standard deviation between sessions divided by the mean between sessions and then averaging these individual CVs to obtain the mean CV [56]. A CV close to 0 indicates a high reproducibility, meaning the measurements have low variability relative to their mean [52, 53, 54].

To assess voxel overlap between sessions, GM and WM segmentations were registered using ANTS [57, 58], followed by a Dice coefficient overlap analysis [59, 60]. Overlaps for each voxel, at each field strength, and location were calculated using the EvaluateSegmentation tool [60]. Dice coefficient overlap using GM and WM masks between sessions at 3 T (GM 0.42, WM 0.53 in paracentral lobule and GM 0.47, WM 0.71 in precentral gyrus) and 7 T (GM 0.32, WM 0.51 in paracentral lobule and GM 0.20, WM 0.39 in precentral gyrus). The overlap is likely impacted by segmentation and registration errors as well as—despite thorough checks—placement of voxels during the repeat sessions. Future studies could employ additional localizers or fiducial markers to improve voxel placement accuracy.

## 3 Results

### 3.1 In Vivo Study

#### 3.1.1 Spectral Quality

**FIGURE 2:**
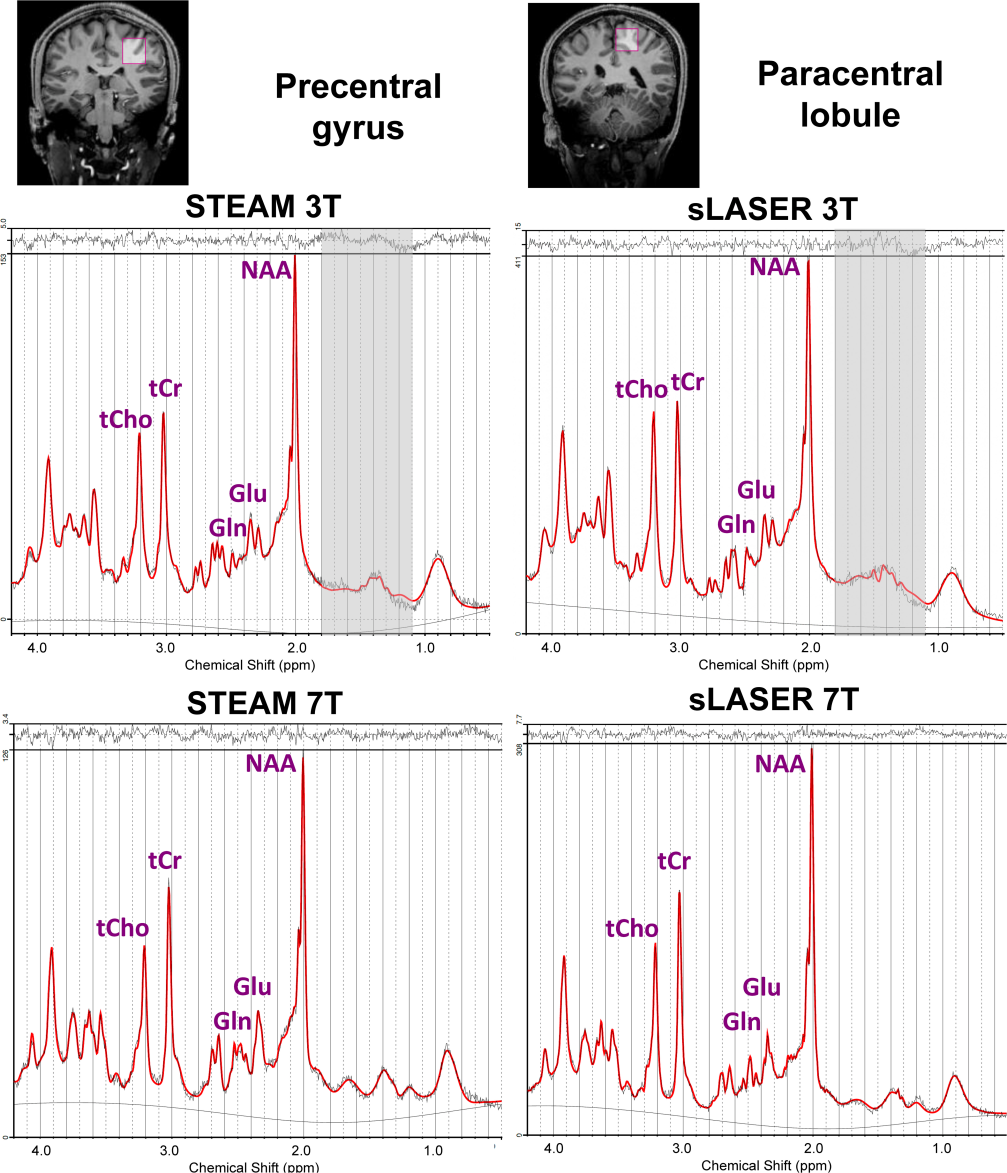
Representative 1H MRS spectra and LCModel fitting results for representative data from one subject measured at 3 T and 7 T from the paracentral lobule and precentral gyrus. 3 T: STEAM (TE = 10 ms, TR = 2 s and NT = 64); sLASER (TE = 28 ms, TR = 2 s and NT = 64). 7 T: STEAM (TE = 8 ms, TR = 8 s and NT = 16); sLASER (TE = 26 ms, TR = 8 s and NT = 16). At 3 T, a gap from 1.1 to 1.85 ppm was used to account for potential lipid contamination and represented a region of lower fitting confidence.

Figure 2 displays representative spectra from one subject obtained from the paracentral lobule and precentral gyrus using STEAM and sLASER sequences at 3 T and 7 T, along with LCModel fits and residuals. Figures S1 and S2 show the average spectra and standard deviation from five volunteers.

##### 3.1.1.1 Field Strength Comparison: 3 T versus 7 T

**FIGURE 3:**
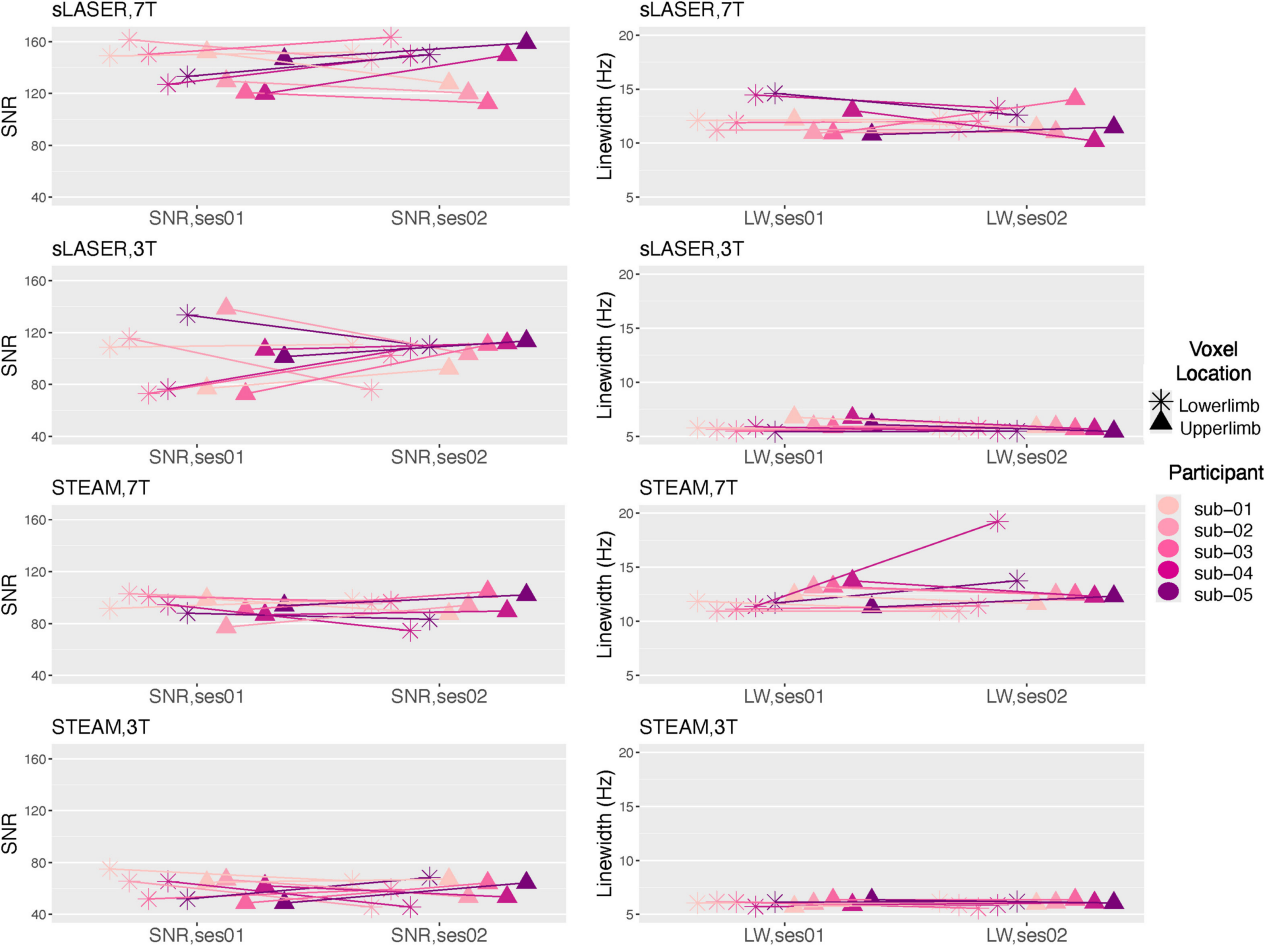
Scatter plots used for comparison of signal‐to‐noise (SNR) total creatine (tCr) and linewidth (Hz) between STEAM and sLASER at both 3 T and 7 T, in the upper limb (precentral gyrus; represented by triangles) and lower limb (paracentral lobule; represented by stars) regions. Each of the five subjects is presented by a different colour, with a connecting line across two sessions to illustrate session‐wise changes.

The comparison between 3 T and 7 T showed significant differences in SNR and linewidth for both STEAM and sLASER sequences (Figure 3 and Table S1). Water FWHM averaged from both voxels was 2.1 times broader at 7 T compared to 3 T, whereas SNR for tCr was 1.5 times higher at 7 T across both sequences even though 16 transients were used at 7 T compared to 64 at 3 T (*p* < 10^−12^). These results showed the superior SNR and broader linewidths achievable with 7 T, aligning with our in vitro finding (Supporting Information).

##### 3.1.1.2 sLASER versus STEAM

A paired *t*‐test revealed significant differences in SNR and linewidth between the sequences at both 3 T and 7 T. At 3 T, sLASER showed significantly higher SNR with a *t* value of 7.6 (*p* < 10^−8^), and at 7 T, the difference was even more pronounced with a *t* value of 11.6 (*p* < 10^−12^), indicating 1.5 times higher SNR compared to STEAM. The water FWHM was significantly broader for the STEAM sequence at both 3 T (*t* value = −2.4, *p* = 0.01) and not significantly at 7 T (*t* value = −0.6, *p* = 0.5) (Figure 3), aligned with our in vitro finding (Supporting Information).

#### 3.1.2 Metabolite Quantifications

**FIGURE 4:**
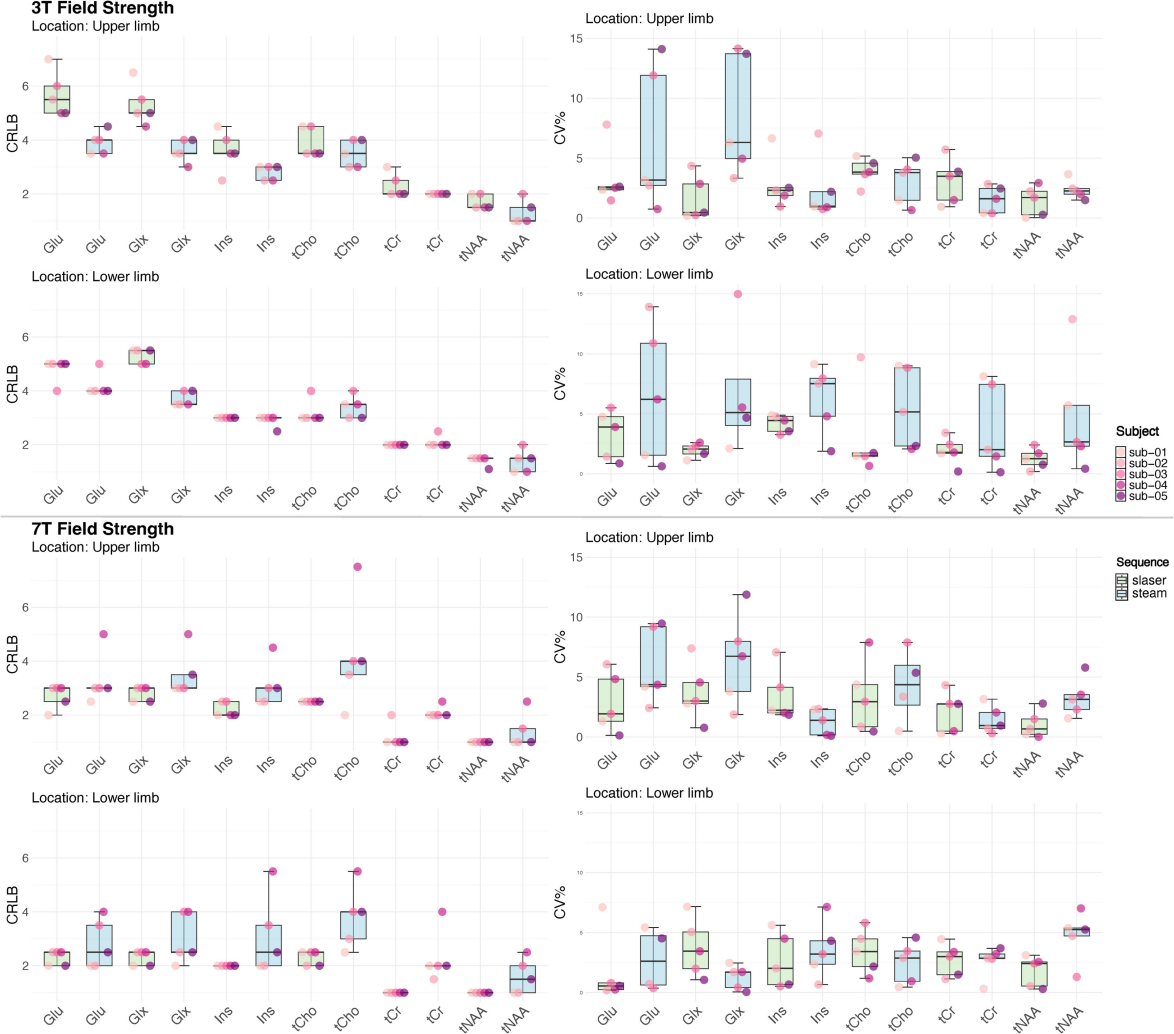
Cramér–Rao lower bound (CRLB) (left side) and intra‐subject coefficient of variations (CVs, %) (right side) from five subjects for high‐concentration metabolite estimates (6 metabolites) in two brain regions (upper limb and lower limb) at 3 T and 7 T for STEAM (blue) and sLASER (green). Most CVs are in the range of 0% and 15%, except for a few outliers. Some metabolites exhibit lower CRLB, and CVs means higher reproducibility than others, leading to a narrower value distribution consequently, represented by smaller boxes in the box plot.

**FIGURE 5:**
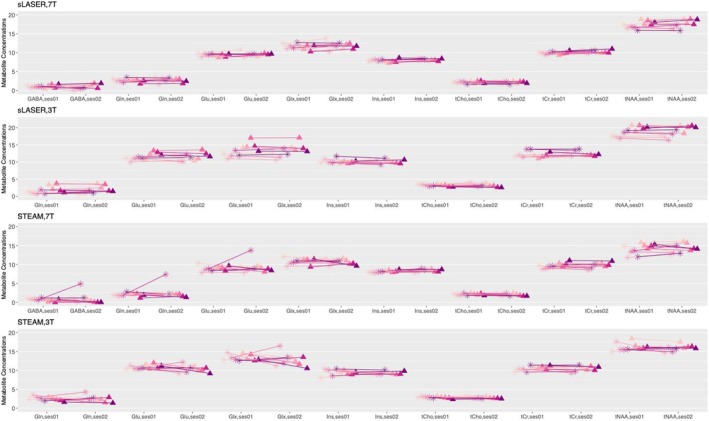
Comparing STEAM and sLASER at both 3 T and 7 T in upper limb (precentral gyrus; represented by triangles) and lower limb (paracentral lobule; represented by stars) regions. The comparison involved five subjects (each represented by a different colour) with a connecting line illustrates the within‐subject change from Session 1 to Session 2. Except for one subject in STEAM (likely due to movement during scan), the concentration estimates were very stable.

Figure 4 provides the individual CRLBs, and Figure 5 shows the metabolite concentration estimates for high‐concentration metabolites at both voxel locations using STEAM and sLASER sequences at 3 T and 7 T. Detailed tables can be found in Tables S2 and S3 for the fitting gap data and in Tables S4 and S5 for the data without the fitting gap. At 3 T, 12 metabolites were reported; GABA, Lac and taurine (Tau) were excluded due to CRLB > 50%. At 7 T, 14 metabolites were reported, with only Asp excluded (Tables S2 and S3).

##### 3.1.2.1 Field Strength Comparison: 3 T versus 7 T

Comparison of CRLBs (Figure 4 and Tables S2 and S3) showed lower values at 7 T compared to 3 T (*t* value = 2.2, *p* = 0.02). The greatest difference in CRLB was observed for glutamine (Gln) (*t* value = 5.5, *p* = 0.000001), whereas metabolites like total NAA (tNAA: NAA + NAAG), total choline (tCho: GPC + PCho) and glutathione (GSH) did not show statistically significant differences in CRLB (*p* > 0.1).

##### 3.1.2.2 sLASER versus STEAM

In overall, the *t*‐test revealed no significant difference in CRLB between sequences (*t* value = −0.01, *p* = 0.9).

##### 3.1.2.3 Precentral Gyrus versus Paracentral Lobule

In both brain regions, the CRLBs for 11 out of 14 metabolites were consistently below 25% across all subjects, sessions and magnetic field strengths. There was no significant difference for CRLBs between voxel locations (*t* value = −1.3, *p* = 0.16).

#### 3.1.3 Reliability and Reproducibility

**FIGURE 6:**
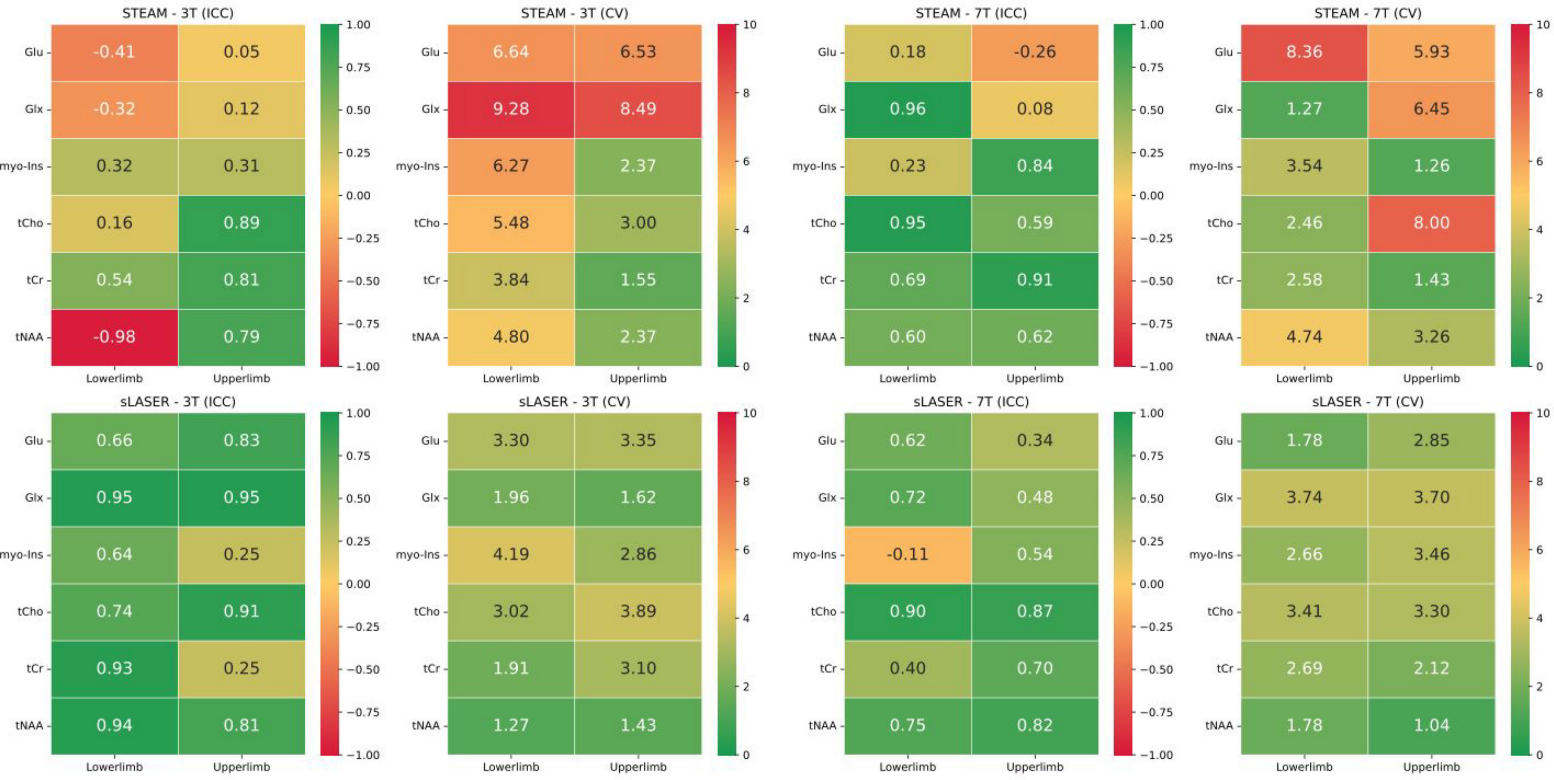
Intraclass correlation coefficient (ICC) and coefficient of variation (CV, %) for both sequences sLASER and STEAM at both 3 T and 7 T for high‐concentration metabolites. ICC reflects reliability as per the following categories: 0.9–0.7 excellent (dark green); 0.7–0.6 good (light green); 0.6–0.4 fair (yellow); < 0.4 poor (orange and red). CV% values represent reproducibility, with values closer to zero indicating better reproducibility, shown in dark green.

Figure 4 shows the intra‐subject CVs, and Figure 6 provides ICC and inter‐subject CVs for seven metabolites at both fields for both locations and sequences. Further details can be found in Tables S2 and S3.

##### 3.1.3.1 Field Strength Comparison: 3 T versus 7 T

In the precentral gyrus, 7 out of 12 metabolites exhibited lower CV% at 7 T compared to 3 T for both sequences, though this difference was not statistically significant (*p* = 0.9). Overall, CV% for estimated metabolite concentrations tended to be higher at 3 T compared to ratio/tCr, but again, the difference was not significant (*t* value = 0.24, *p* = 0.81).

##### 3.1.3.2 Sequence Comparison: sLASER versus STEAM

At 3 T in the precentral gyrus for estimated metabolite concentrations, sLASER exhibited higher CV% for 6 out of 12 metabolites but better reliability (higher ICC) for 10 out of 12 metabolites. STEAM showed better reliability and reproducibility for the remaining metabolites. In the paracentral lobule at 3 T, sLASER demonstrated lower CV% and higher ICC for 10 out of 12 metabolites (see Tables S2 and S3).

At 7 T for estimated metabolite concentrations, sLASER showed excellent reproducibility (lower CV%), for most metabolites, except for Lac, PE, myo‐Ins and Tau, and demonstrated higher ICC for 9 out of 14 metabolites, indicating superior reliability at both locations. For the remaining metabolites, STEAM showed good to excellent reliability (ICC > 0.6; see Figure 6). Notably, at 7 T, sLASER showed lower CV% for GABA (21% in the paracentral lobule and 8% in the precentral gyrus) compared to STEAM (30% and 23%, respectively).

##### 3.1.3.3 Voxel Location Comparison: Precentral Gyrus versus Paracentral Lobule

In the precentral gyrus, high‐concentration metabolites with CRLB ≤ 10% had mean CV% values below 8%, indicating excellent reproducibility, with tNAA showing a minimum CV% of 1% and Glu a maximum of 8%. In the paracentral lobule, similarly, metabolites with low CRLBs (≤ 10%) demonstrated excellent reproducibility, with tNAA at 1% and Glx (Glu + Gln) at 10%.

## 4 Discussion

This study evaluated the reliability and reproducibility of brain metabolite concentrations measured by STEAM and sLASER sequences at 3 T and 7 T in both phantom and in vivo in two different areas of the human brain: the precentral gyrus and paracentral lobule. Our results showed the sLASER sequence exhibited superior SNR, reliability and reproducibility, making it preferable for longitudinal studies. Additionally, whereas the 7 T scanner offers higher SNR and spectral resolution, allowing for better separation and quantification, 3 T scanners still provide excellent or good reliability for most metabolites for the same acquisition time, making it a viable option when 7 T is unavailable.

### 4.1 Comparison With Previous Studies

CRLB and metabolite concentration have been used in four previous studies for field strength comparisons [14, 16, 61, 62] with two studies that investigated reproducibility by reporting CV [5, 63]. There were only two studies that compared sequences at 7 T [11, 25] and one at 4 T [37]. Other studies have examined the reproducibility at 3 T using STEAM [17] and sLASER [13] and at 7 T using STEAM [9, 15, 19, 56, 64] and sLASER [10]. Our acquisition parameters closely resembled those of Deelchand et al. [13] for sLASER at 3 T and Terpstra et al. [5] for sLASER at both 3 T and 7 T using the same scanner (Siemens) but examined different brain regions, head coils, voxel sizes and scan intervals. Notably, our study did not use dielectric pads, resulting in a longer TR at 7 T due to reaching the SAR limit, suggesting that other researchers may have benefited from improved transmit field efficiency [15, 65]. Whereas Bell et al. [20] focused on repeatability within the same session, our study goes further by comparing both reproducibility and reliability over multiple sessions, providing more insight into how these sequences perform over time in a real‐world setting.

### 4.2 Spectral Quality

We successfully obtained high‐quality spectra by employing both STEAM and sLASER sequences across two fields evidenced by high SNR, narrow linewidths and low CRLBs. Although the voxel locations used in this study are not directly comparable with previous literature, our results align well with previously reported SNR and linewidths in other brain regions [14]. We observed an increase in spectral linewidth (2.2 times higher at 7 T compared to 3 T), which corresponded with an increase in the spectral dispersion of singlet resonances. We also noted an increase in SNR by 45% from 3 to 7 T, though this is lower than the expected supralinear increase predicted by theory, where SNR is anticipated to scale as SNR ∼ B_0_^1.65^ [66]. This discrepancy may be due to the use of different receive coils—64 channels at 3 T versus 32 channels at 7 T—making direct SNR comparisons between systems challenging. Moreover, we observed 80% higher SNR at 7 T in our phantom study; further details can be found in the Supporting Information. Moreover, we noticed the effect of T_1_ relaxation times when comparing the SNR at 3 T and 7 T. At 7 T, a longer TR of 8 s allows for more complete magnetization recovery, resulting in higher SNR. Based on T_1_ values for tCr at 3 T and 7 T, the tCr SNR loss due to T_1_ saturation effects at 3 T with a TR of 2 s is 19% in WM and 25% in GM compared to 7 T.

Interestingly, the SNR in our in vivo experiments was slightly higher than in previous studies [14, 22]. This could be attributed to our voxel placements [67], effective shimming using the FAST (EST)MAP [68], and our relatively large voxel size. As a result, we were able to extend the sensitivity evaluation to additional metabolites that were reliably detected, including GABA, Glu, Gln, GSH and Tau (Tables S2 and S3). As anticipated, the STEAM sequence had a noticeably lower SNR than sLASER when used in similar scan times with a similar number of transients across both fields [11], both in vivo and in vitro. In addition to the lower available signal in STEAM, the observed 50% higher SNR for sLASER at 3 T compared to STEAM may also be influenced by differences in sequence profiles due to the use of different RF pulses in each sequence.

### 4.3 Reliability and Reproducibility

Reliability and reproducibility may vary by voxel location due to differences in coil placement and measurement conditions like shim and outer volume contamination [14, 69]. Reporting both ICC and CV% offers a more comprehensive assessment of a given sequence's performance. When examining the reliability and reproducibility of MRS techniques and making comparisons between studies, several elements come into play. One is the quantification factor. In this research, we employed the normalization of metabolite ratios to tCr and concentrations estimates for the computation of both CV% and ICC. Generally, metabolites exhibited a marginally lower CV% when their ratio to tCr was used compared to the relaxation and partial volume corrected concentrations, indicating enhanced reproducibility with the internal tCr reference compared to external references like water. Another reason for the lower CV when examining the metabolite ratio to tCr is the correction of relaxation time. As anticipated, high‐concentration metabolites and a CRLB of 10 or less exhibited excellent reproducibility with mean CVs of 8% and a reliability range of 0.6–0.9 in both regions under study. Low‐concentration metabolites, which had low SNR, and those with signals that overlapped with other metabolites, tended to have slightly higher mean CVs. Despite the use of only 16 transients at 7 T, a high level of reproducibility was achieved. When compared to the commonly used STEAM sequence, the sLASER sequence proved to be more reliable at 7 T in both regions for most metabolites, except for Glu, myo‐Ins and Tau. At 3 T, STEAM was a more reliable method for determining GSH, myo‐Ins and Tau. The mean CVs reported in this study were, on average, lower than those reported in previous studies [9, 10, 56]. The lower CVs in this study could be attributed to factors such as voxel locations and size, shorter scan times at both scanners, number of participants and/or the use of young, healthy adults as subjects. Additionally, the basis set used in this study included 19 metabolites, which helped prevent systematic bias and errors in fitting [70].

In our study, at both fields, sLASER demonstrated good to excellent reliability (high ICC) for the majority of metabolites, with the exceptions of myo‐Ins (−0.1), Lac and phosphoethanolamine (PE) (0.2) at 7 T and GABA (−0.2) and GSH (−0.09) at 3 T, suggesting reliable measurements across sessions. The excellent reliability observed for metabolites such as tCho, tCr, Glx and tNAA in our study can be attributed to their biochemical stability, clear spectral signals and the robustness of the acquisition techniques used. These metabolites are known for their stability in the brain and exhibit well‐defined peaks in the MRS spectrum, making them easier to quantify reliably. These results suggest that sLASER can provide more reliable and reproducible between sessions. Our findings are in line with previous work [11] that shows ICCs of around 0.6 for sLASER at 7 T (12‐metabolite fit result).

Another advantage of sLASER is related to the spatial accuracy of voxel localization, particularly in the presence of CSDE at high field strengths, as adiabatic full‐passage (AFP) pulses like gradient offset independent adiabatic (GOIA)‐WURST can achieve high bandwidths, thereby minimizing CSDE [27, 71]. For the given slice‐selective 90° pulse and AFP GOIA‐WURST refocusing pulses bandwidths in Table 1, the CSDEs were as follows: for STEAM 4.9% per ppm at both 3 T and 7 T and for sLASER, 3% per ppm at 3 T and 4.9% at 7 T and significantly lower for the AFP refocusing pulses 0.27% per ppm at 3 T and 0.66% per ppm at 7 T.

Despite using the same approach for quantifying metabolites, we noticed a minor bias in the estimation of certain metabolite concentrations (i.e., Gln, Glx and tNAA) between STEAM and sLASER. This could be due to the lack of consideration for the differentiation between sLASER and STEAM/PRESS relaxation times, which can influence the precision of reported concentration estimate. This is because the numerous refocusing pulses in sLASER function as Carr–Purcell (CP) pulse trains to minimize *J*‐evolution and extend the apparent transverse (T_2_) relaxation times. CP pulse trains can also affect the T_2_ relaxation of water, potentially influencing the metabolite concentration estimates [27, 71, 72]. For corrections related to tissue‐specific relaxation, Osprey utilized the T_1_ and T_2_ relaxations of metabolites at 3 T and 7 T, as reported in the literature [31, 73, 74, 75, 76, 77, 78, 79, 80]. However, the literature is sparser for 7 T, specifically for sLASER [31, 80]. It is important to acknowledge that metabolite concentration estimates are influenced by the acquisition methods and field strengths used. These values are not absolute and can vary depending on various factors, including sequence parameters, voxel positioning and field inhomogeneities. Therefore, careful interpretation is needed when comparing data across sequences and field strengths.

Our findings have significant implications for tracking the progression of brain disorders, such as neurodegenerative diseases. Despite the lower SNR at 3 T, we found that 3 T is effective for quantifying Glx, tCho, myo‐Ins and tNAA in the precentral gyrus and paracentral lobule, as the CV% does not exceed 10% (as shown in Figure 6).

### 4.4 Limitations

This study has several limitations. Firstly, our sample size was smaller compared to other reproducibility studies. This limitation is largely due to the extensive scanning protocol, which required approximately 5 h per participant across multiple sessions, and including scans at two field strengths, with two sequences and two voxel locations. Despite this, the study's design yielded a robust dataset of 80, providing meaningful results for reliability and reproducibility analyses. Secondly, at 3 T, a fitting gap between 1.1 and 1.85 ppm was applied to address lipid contamination observed in some sLASER datasets (Figures S1 and S2), which was visible particularly in the precentral gyrus region. This artefact may be due to slight differences in spoiler gradient amplitude for sLASER (37 mT/m at 3 T vs. 40 mT/m at 7 T). Although the fitting gap improved fitting stability, it may impact detection of metabolites in this spectral range and highlight the sensitivity of lipid suppression to the sequence design and anatomical location. Thirdly, although all scans were conducted within a 5–9 days' interval, we did not strictly regulate the time of day at which scans were performed, which may introduce variability due to potential diurnal fluctuations in metabolite concentrations; previous studies have reported mixed findings on this issue [15, 81, 82]. Fourthly, the longer TR required at 7 T for SAR safety restricts our ability to optimize SNR per unit time at this field strength, representing a practical trade‐off in ultra‐high‐field MRS studies. Fifthly, to mitigate motion, we selected younger participants and used padding to restrict movement. Lastly, the uniformity of the phantom in our study minimizes the observable effects of CSDE, which may have a more significant impact in biological tissues due to non‐uniform metabolite distribution.

## 5 Conclusion

Based on the data and findings from this study, it can be concluded that the sLASER sequence demonstrated superior SNR, along with enhanced reliability and reproducibility. This was observed in both the precentral gyrus and paracentral lobule regions and was achieved within a practical acquisition time. These attributes make sLASER the preferred sequence for longitudinal studies that investigate changes in metabolite concentrations, where consistent and reliable data over time is crucial.

Furthermore, when available, the use of a 7 T scanner is recommended due to its higher SNR, which can provide more detailed and accurate data. However, it is important to note that 3 T scanners also exhibit excellent or good reliability for most metabolites. This makes it a viable option when a 7 T scanner is not accessible.

In summary, the choice of sequence and scanner can significantly impact data quality in longitudinal MRS studies, and this study should help to guide researchers in making informed decisions to ensure the reliability and reproducibility of their results.

## Author Contributions

**Zeinab Eftekhari:** conceptualization, methodology, software, validation, investigation, formal analysis, resources, visualization, writing – original draft. **Thomas B. Shaw** and **Wolfgang Bogner:** conceptualization, software, validation, investigation, formal analysis, supervision, reviewing and editing of manuscript. **Dinesh K. Deelchand and Małgorzata Marjańska:** methodology, validation, investigation, resources, reviewing and editing of manuscript. **Markus Barth:** conceptualization, validation, investigation, resources, supervision, reviewing and editing of manuscript, funding acquisition. All authors contributed to the article and approved the submitted version.

## Ethics Statement

All participants provided written informed consent, and the study was approved by the local Human Research Ethics Committee.

## Conflicts of Interest

The authors declare no conflicts of interest.

## Supporting information


**Table S1** tCr SNR and water linewidth for both sequences at both fields in two different locations.
**Table S2.** Precentral gyrus metabolite concentration estimate, ratios to tCr, reliability and reproducibility measures.
**Table S3.** Paracentral lobule metabolite concentration estimate, ratios to tCr, reliability and reproducibility measures.
**Table S4.** Precentral gyrus metabolite concentration estimate, ratios to tCr, reliability and reproducibility measures at 3 T without using the ppm gap.
**Table S5.** Paracentral lobule metabolite concentration estimate, ratios to tCr, reliability and reproducibility measures at 3 T without using the ppm gap.
**Table S6.** Metabolite ratios to tCr and reproducibility measures from phantom scans.
**Table S7.** The MRSinMRS checklist.
**Figure S1.** Average (full line) and standard deviation (shaded area) of individual spectra are shown for 5 volunteers at 3 T for two locations (upper limb and lower limb) from Session 1 (left side) and Session 2 (right side) for both sequences (sLASER and STEAM). A gap from 1.1 to 1.85 ppm was used to account for potential lipid contamination and represented a region of lower fitting confidence.
**Figure S2.** Average (full line) and standard deviation (shaded area) of individual spectra are shown for 5 volunteers at 7 T for two locations (upper limb and lower limb) from Session 1 (left side) and Session 2 (right side) for both sequences (sLASER and STEAM).

## Data Availability

All code for this experiment and CSVs of all post‐processed MRS metabolite are provided in https://github.com/Zeinabeftekhari/SVS_STEAM‐sLASER_3T‐7T.git. Due to the size of the raw Siemens files and confidentiality of basis sets, we are unable to share raw Siemens data.
