# Supplementary material for: Reliability and Reproducibility of Metabolite Quantification Using 1H MRS in the Human Brain at 3 T and 7 T
Source: NMR Biomed. 2025 Jul 8;38(8):e70087. doi: 10.1002/nbm.70087 (PMC12235355; doi:10.1002/nbm.70087)
Supplement: Supplementary file 1 — Table S1 tCr SNR and water linewidth for both sequences at both fields in two different locations. Table S2. Precentral gyrus metabolite concentration estimate, ratios to tCr, reliability and reproducibility measures. Table S3. Paracentral lobule metabolite concentration estimate, ratios to tCr, reliability and reproducibility measures. Table S4. Precentral gyrus metabolite concentration estimate, ratios to tCr, reliability and reproducibility measures at 3 T without using the ppm gap. Table S5. Paracentral lobule metabolite concentration estimate, ratios to tCr, reliability and reproducibility measures at 3 T without using the ppm gap. Table S6. Metabolite ratios to tCr and reproducibility measures from phantom scans. Table S7. The MRSinMRS checklist. Figure S1. Average (full line) and standard deviation (shaded area) of individual spectra are shown for 5 volunteers at 3 T for two locations (upper limb and lower limb) from Session 1 (left side) and Session 2 (right side) for both sequences (sLASER and STEAM). A gap from 1.1 to 1.85 ppm was used to account for potential lipid contamination and represented a region of lower fitting confidence. Figure S2. Average (full line) and standard deviation (shaded area) of individual spectra are shown for 5 volunteers at 7 T for two locations (upper limb and lower limb) from Session 1 (left side) and Session 2 (right side) for both sequences (sLASER and STEAM). [file NBM-38-e70087-s001.docx]

**­12. Supplementary Materials**

*Table S1: tCr SNR, water linewidth for both sequences at both fields in two different locations*

| ***tCr SNR*** | ***Water FWHM (Hz)*** | ***Sequence*** | ***Field strength*** | ***Location*** |
| --- | --- | --- | --- | --- |
| 99.7 ± 24 | 5.9 ± 0.4 | sLASER | 3T | Precentral gyrus |
| 59.4 ± 7 | 5.8 ± 0.2 | STEAM | 3T | Precentral gyrus |
| 132.7 ± 17 | 11.8 ± 1.1 | sLASER | 7T | Precentral gyrus |
| 92.5 ± 7 | 12.4 ± 0.6 | STEAM | 7T | Precentral gyrus |
| 101.3 ± 18 | 5.6 ± 0.1 Hz | sLASER | 3T | Paracentral lobule |
| 59.4 ± 9 | 5.9 ± 0.2 | STEAM | 3T | Paracentral lobule |
| 148.0 ± 10 | 12.5 ± 1.1 | sLASER | 7T | Paracentral lobule |
| 92.5 ± 8 | 12.3 ± 2.4 | STEAM | 7T | Paracentral lobule |

*Table S2: Precentral gyrus metabolite concentration estimate, ratios to tCr, reliability and reproducibility measure*s.

| ***Metabolite*** | ***Sequence*** | ***Field Strength*** | ***Mean concentration estimate (std dev.)*** | | ***Mean CRLB (%)*** | ***Mean CV using concentration estimate (%)*** | ***ICC using concentration estimate*** | ***Mean /tCr (std dev.)*** | | ***Mean CV tCr (%)*** | ***ICC tCr*** | ***N*** |
| --- | --- | --- | --- | --- | --- | --- | --- | --- | --- | --- | --- | --- |
| ***Asc*** | STEAM | 3 | 1.2 | 0.4 | 34 | 11.30 | 0.30 | 0.1 | 0.04 | 9.70 | 0.26 | 5 |
|  | STEAM | 7 | 1.8 | 0.3 | 21 | 17.24 | -0.02 | 0.1 | 0.03 | 17.86 | -0.20 | 10 |
|  | sLASER | 3 | 1.2 | 0.0 | 37 | 26.00 | 0.38 | 0.1 | 0.01 | 13.06 | 0.56 | 5 |
|  | sLASER | 7 | 1.8 | 0.3 | 14 | 9.17 | 0.58 | 0.1 | 0.03 | 10.25 | 0.44 | 10 |
| ***Asp*** | STEAM | 3 | 3.2 | 0.4 | 12 | 10.15 | 0.37 | 0.3 | 0.05 | 10.40 | 0.53 | 10 |
|  | sLASER | 3 | 3.2 | 0.6 | 24 | 14.54 | 0.39 | 0.2 | 0.05 | 10.87 | 0.51 | 10 |
| ***tCr*** | STEAM | 3 | 10.6 | 0.4 | 2 | 1.55 | 0.81 | 1.0 | 0.00 | 0.00 | NA | 10 |
|  | STEAM | 7 | 10.1 | 0.5 | 2 | 1.43 | 0.91 | 1.0 | 0.00 | 0.00 | NA | 10 |
|  | sLASER | 3 | 11.9 | 0.4 | 2 | 3.10 | 0.25 | 1.0 | 0.00 | 0.00 | NA | 10 |
|  | sLASER | 7 | 10.0 | 0.4 | 1 | 2.12 | 0.70 | 1.0 | 0.00 | 0.00 | NA | 10 |
| ***GABA*** | STEAM | 3 | 1.1 | 0.2 | 31 | 18.10 | 0.78 | 0.1 | 0.02 | 16.91 | 0.78 | 5 |
|  | STEAM | 7 | 0.7 | 0.0 | 30 | 23.07 | 0.19 | 0.0 | 0.00 | 19.98 | 0.26 | 3 |
|  | sLASER | 7 | 1.3 | 0.4 | 25 | 8.18 | 0.71 | 0.1 | 0.04 | 5.84 | 0.69 | 9 |
| ***Gln*** | STEAM | 3 | 2.1 | 0.4 | 17 | 17.38 | 0.45 | 0.2 | 0.05 | 16.63 | 0.51 | 10 |
|  | STEAM | 7 | 1.9 | 0.4 | 15 | 10.63 | 0.71 | 0.1 | 0.04 | 10.70 | 0.79 | 10 |
|  | sLASER | 3 | 2.4 | 1.0 | 21 | 9.62 | 0.96 | 0.2 | 0.05 | 13.58 | 0.40 | 10 |
|  | sLASER | 7 | 2.5 | 0.4 | 10 | 6.71 | 0.79 | 0.2 | 0.04 | 5.71 | 0.88 | 10 |
| ***Glu*** | STEAM | 3 | 10.6 | 0.5 | 3 | 6.53 | 0.05 | 1.0 | 0.05 | 5.84 | 0.13 | 10 |
|  | STEAM | 7 | 8.9 | 0.3 | 3 | 5.93 | -0.26 | 0.8 | 0.05 | 5.31 | 0.20 | 10 |
|  | sLASER | 3 | 12.0 | 1.0 | 5 | 3.35 | 0.83 | 1.0 | 0.06 | 7.92 | -0.11 | 10 |
|  | sLASER | 7 | 9.3 | 0.3 | 2 | 2.85 | 0.34 | 0.9 | 0.03 | 2.13 | 0.58 | 10 |
| ***Glx*** | STEAM | 3 | 12.8 | 0.8 | 3 | 8.49 | 0.12 | 1.2 | 0.1 | 7.75 | 0.32 | 10 |
|  | STEAM | 7 | 10.7 | 0.6 | 3 | 6.45 | 0.08 | 1.0 | 0.09 | 5.84 | 0.48 | 10 |
|  | sLASER | 3 | 14.5 | 1.4 | 5 | 1.62 | 0.95 | 1.3 | 0.07 | 2.26 | 0.73 | 10 |
|  | sLASER | 7 | 11.4 | 0.5 | 2 | 3.70 | 0.48 | 1.1 | 0.07 | 2.69 | 0.76 | 10 |
| ***tCho*** | STEAM | 3 | 2.7 | 0.2 | 3 | 3.00 | 0.89 | 0.2 | 0.03 | 2.98 | 0.90 | 10 |
|  | STEAM | 7 | 2.0 | 0.2 | 4 | 8.00 | 0.59 | 0.2 | 0.03 | 7.65 | 0.73 | 10 |
|  | sLASER | 3 | 3.0 | 0.4 | 3 | 3.89 | 0.91 | 0.2 | 0.03 | 5.87 | 0.70 | 10 |
|  | sLASER | 7 | 2.2 | 0.2 | 2 | 3.30 | 0.87 | 0.2 | 0.03 | 5.12 | 0.85 | 10 |
| ***GSH*** | STEAM | 3 | 1.6 | 0.2 | 11 | 4.05 | 0.73 | 0.1 | 0.02 | 4.85 | 0.74 | 10 |
|  | STEAM | 7 | 0.8 | 0.1 | 20 | 18.10 | -0.08 | 0.08 | 0.01 | 16.97 | 0.12 | 10 |
|  | sLASER | 3 | 2.3 | 0.5 | 13 | 3.69 | 0.96 | 0.2 | 0.02 | 14.92 | -0.31 | 10 |
|  | sLASER | 7 | 1.5 | 0.1 | 9 | 5.48 | 0.59 | 0.1 | 0.01 | 3.85 | 0.84 | 10 |
| ***myo-Ins*** | STEAM | 3 | 9.3 | 0.3 | 2 | 2.37 | 0.31 | 0.8 | 0.04 | 2.61 | 0.60 | 10 |
|  | STEAM | 7 | 8.3 | 0.3 | 3 | 1.26 | 0.84 | 0.8 | 0.02 | 1.70 | 0.67 | 10 |
|  | sLASER | 3 | 9.9 | 0.3 | 3 | 2.86 | 0.25 | 0.8 | 0.03 | 4.69 | -0.41 | 10 |
|  | sLASER | 7 | 7.8 | 0.3 | 2 | 3.46 | 0.54 | 0.7 | 0.01 | 2.56 | -0.52 | 10 |
| ***Lac*** | STEAM | 7 | 1.9 | 0.8 | 10 | 18.39 | 0.87 | 0.2 | 0.08 | 19.13 | 0.86 | 10 |
|  | sLASER | 7 | 1.6 | 0.5 | 9 | 20.19 | 0.77 | 0.2 | 0.08 | 19.06 | 0.69 | 9 |
| ***tNAA*** | STEAM | 3 | 16.4 | 0.8 | 1 | 2.37 | 0.79 | 1.5 | 0.1 | 2.00 | 0.86 | 10 |
|  | STEAM | 7 | 14.9 | 0.7 | 1 | 3.26 | 0.62 | 1.4 | 0.10 | 2.75 | 0.83 | 10 |
|  | sLASER | 3 | 19.9 | 0.8 | 1 | 1.43 | 0.81 | 1.7 | 0.09 | 1.24 | 0.90 | 10 |
|  | sLASER | 7 | 18.2 | 0.6 | 1 | 1.04 | 0.82 | 1.8 | 0.08 | 0.41 | 0.98 | 10 |
| ***PE*** | STEAM | 3 | 1.4 | 0.2 | 35 | 20.71 | 0.21 | 0.1 | 0.02 | 21.24 | 0.30 | 5 |
|  | STEAM | 7 | 1.4 | 0.3 | 32 | 21.86 | 0.74 | 0.1 | 0.05 | 23.26 | 0.71 | 4 |
|  | sLASER | 3 | 2.8 | 0.4 | 28 | 12.94 | 0.85 | 0.2 | 0.05 | 16.99 | 0.87 | 7 |
|  | sLASER | 7 | 2.0 | 0.5 | 18 | 23.15 | 0.27 | 0.2 | 0.09 | 48.97 | 0.38 | 9 |
| ***sIns*** | STEAM | 3 | 0.5 | 0.1 | 13 | 16.91 | 0.52 | 0.05 | 0.01 | 17.76 | 0.56 | 10 |
|  | STEAM | 7 | 0.2 | 0.1 | 38 | 9.21 | 0.97 | 0.02 | 0.01 | 9.19 | 0.97 | 9 |
|  | sLASER | 3 | 0.4 | 0.1 | 27 | 27.65 | 0.55 | 0.04 | 0.02 | 23.35 | 0.75 | 9 |
|  | sLASER | 7 | 0.2 | 0.1 | 19 | 17.84 | 0.77 | 0.03 | 0.01 | 16.80 | 0.78 | 10 |
| ***Tau*** | STEAM | 7 | 2.6 | 0.2 | 13 | 7.13 | 0.51 | 0.2 | 0.03 | 7.87 | 0.63 | 10 |
|  | sLASER | 7 | 2.2 | 0.2 | 8 | 9.54 | 0.37 | 0.2 | 0.02 | 9.81 | 0.29 | 10 |

*Table S3: Paracentral lobule metabolite concentration estimate, ratios to tCr, reliability and reproducibility measures*

| ***Metabolite*** | ***Sequence*** | ***Field Strength*** | ***Mean concentration estimate (std dev.)*** | | ***Mean CRLB (%)*** | ***Mean CV concentration estimate (%)*** | ***ICC using concentration estimate*** | ***Mean /tCr (std dev.)*** | | ***Mean CV tCr (%)*** | ***ICC tCr*** | ***N*** |
| --- | --- | --- | --- | --- | --- | --- | --- | --- | --- | --- | --- | --- |
| ***Asc*** | STEAM | 3 | 1.7 | 0.4 | 24 | 14.27 | -0.53 | 0.1 | 0.04 | 12.86 | -0.71 | 5 |
|  | STEAM | 7 | 2.9 | 0.1 | 10 | 4.42 | -0.08 | 0.3 | 0.02 | 4.21 | 0.01 | 10 |
|  | sLASER | 3 | 3.8 | 0.5 | 12 | 11.60 | 0.45 | 0.3 | 0.04 | 7.58 | 0.63 | 5 |
|  | sLASER | 7 | 2.3 | 0.3 | 11 | 11.81 | 0.53 | 0.2 | 0.04 | 9.73 | 0.69 | 10 |
| ***Asp*** | STEAM | 3 | 3.2 | 0.6 | 14 | 17.24 | 0.33 | 0.3 | 0.06 | 19.53 | 0.19 | 10 |
|  | sLASER | 3 | 3.7 | 0.5 | 17 | 6.65 | 0.79 | 0.3 | 0.03 | 7.61 | 0.35 | 10 |
| ***tCr*** | STEAM | 3 | 10.2 | 0.6 | 2 | 3.84 | 0.54 | 1.0 | 0.00 | 0.00 | NA | 10 |
|  | STEAM | 7 | 9.3 | 0.4 | 2 | 2.58 | 0.69 | 1.0 | 0.00 | 0.00 | NA | 10 |
|  | sLASER | 3 | 12.6 | 0.9 | 2 | 1.91 | 0.93 | 1.0 | 0.00 | 0.00 | NA | 10 |
|  | sLASER | 7 | 10.1 | 0.2 | 1 | 2.69 | 0.40 | 1.0 | 0.00 | 0.00 | NA | 10 |
| ***GABA*** | STEAM | 3 | 1.5 | 0.8 | 29 | 43.40 | -0.36 | 0.1 | 0.08 | 47.41 | -0.28 | 3 |
|  | STEAM | 7 | 1.2 | 0.8 | 21 | 30.14 | -0.12 | 0.1 | 0.09 | 31.43 | -0.13 | 3 |
|  | sLASER | 7 | 0.9 | 0.1 | 28 | 21.71 | 0.35 | 0.09 | 0.01 | 24.60 | 0.31 | 9 |
| ***Gln*** | STEAM | 3 | 2.6 | 0.5 | 15 | 19.45 | -0.06 | 0.2 | 0.06 | 20.58 | 0.06 | 10 |
|  | STEAM | 7 | 2.6 | 1.1 | 10 | 24.06 | -0.07 | 0.2 | 0.1 | 25.14 | -0.10 | 10 |
|  | sLASER | 3 | 1.2 | 0.3 | 36 | 9.41 | 0.66 | 0.1 | 0.04 | 3.17 | 0.66 | 10 |
|  | sLASER | 7 | 2.7 | 0.5 | 8 | 12.82 | 0.60 | 0.2 | 0.05 | 11.21 | 0.61 | 10 |
| ***Glu*** | STEAM | 3 | 10.5 | 0.5 | 4 | 6.64 | -0.41 | 1.0 | 0.08 | 8.99 | -0.19 | 10 |
|  | STEAM | 7 | 9.2 | 1.3 | 2 | 8.36 | 0.18 | 0.9 | 0.1 | 8.98 | 0.05 | 10 |
|  | sLASER | 3 | 11.0 | 0.6 | 4 | 3.30 | 0.66 | 0.9 | 0.07 | 6.24 | 0.12 | 10 |
|  | sLASER | 7 | 9.5 | 0.4 | 2 | 1.78 | 0.62 | 0.9 | 0.04 | 2.53 | 0.68 | 10 |
| ***Glx*** | STEAM | 3 | 13.2 | 0.9 | 3 | 9.28 | -0.32 | 1.3 | 0.1 | 11.41 | -0.08 | 10 |
|  | STEAM | 7 | 10.7 | 0.8 | 3 | 1.27 | 0.96 | 1.2 | 0.2 | 11.99 | -0.05 | 10 |
|  | sLASER | 3 | 12.1 | 1.0 | 5 | 1.96 | 0.95 | 1.1 | 0.1 | 3.64 | 0.87 | 10 |
|  | sLASER | 7 | 11.9 | 0.9 | 2 | 3.74 | 0.72 | 1.1 | 0.08 | 2.50 | 0.81 | 10 |
| ***tCho*** | STEAM | 3 | 2.8 | 0.1 | 3 | 5.48 | 0.16 | 0.2 | 0.03 | 2.28 | 0.93 | 10 |
|  | STEAM | 7 | 2.0 | 0.2 | 3 | 2.46 | 0.95 | 0.2 | 0.03 | 4.67 | 0.85 | 10 |
|  | sLASER | 3 | 3.3 | 0.3 | 3 | 3.02 | 0.74 | 0.2 | 0.03 | 2.44 | 0.92 | 10 |
|  | sLASER | 7 | 2.0 | 0.2 | 2 | 3.41 | 0.90 | 0.2 | 0.03 | 4.23 | 0.86 | 10 |
| ***GSH*** | STEAM | 3 | 1.2 | 0.1 | 18 | 11.53 | 0.41 | 0.1 | 0.04 | 10.98 | 0.49 | 10 |
|  | STEAM | 7 | 1.3 | 0.1 | 10 | 11.63 | 0.49 | 0.1 | 0.01 | 10.67 | 0.42 | 10 |
|  | sLASER | 3 | 1.8 | 0.3 | 10 | 17.47 | -0.09 | 0.1 | 0.03 | 15.61 | 0.76 | 10 |
|  | sLASER | 7 | 0.8 | 0.2 | 16 | 6.09 | 0.54 | 0.09 | 0.02 | 6.45 | 0.46 | 10 |
| ***myo-Ins*** | STEAM | 3 | 9.4 | 0.6 | 2 | 6.27 | 0.32 | 0.9 | 0.05 | 2.49 | 0.73 | 10 |
|  | STEAM | 7 | 8.1 | 0.2 | 3 | 3.54 | 0.23 | 0.8 | 0.04 | 4.25 | 0.12 | 10 |
|  | sLASER | 3 | 10.3 | 0.6 | 3 | 4.19 | 0.64 | 0.8 | 0.03 | 1.82 | 0.65 | 10 |
|  | sLASER | 7 | 8.1 | 0.1 | 2 | 2.66 | -0.11 | 0.8 | 0.03 | 1.29 | 0.84 | 10 |
| ***Lac*** | STEAM | 7 | 1.0 | 0.2 | 12 | 13.12 | 0.55 | 0.1 | 0.03 | 10.94 | 0.53 | 10 |
|  | sLASER | 7 | 1.1 | 0.3 | 12 | 27.67 | 0.26 | 0.1 | 0.04 | 27.03 | 0.27 | 9 |
| ***tNAA*** | STEAM | 3 | 15.6 | 0.3 | 1 | 4.80 | 0.89 | 1.5 | 0.1 | 5.50 | 0.12 | 10 |
|  | STEAM | 7 | 13.3 | 0.9 | 1 | 4.74 | 0.60 | 1.4 | 0.1 | 4.18 | 0.57 | 10 |
|  | sLASER | 3 | 17.8 | 1.0 | 1 | 1.27 | 0.94 | 1.4 | 0.05 | 1.80 | 0.70 | 10 |
|  | sLASER | 7 | 16.7 | 0.6 | 1 | 1.78 | 0.75 | 1.6 | 0.07 | 2.21 | 0.68 | 10 |
| ***PE*** | sLASER | 3 | 3.5 | 0.2 | 22 | 5.94 | 0.41 | 0.2 | 0.05 | 8.66 | 0.69 | 7 |
|  | sLASER | 7 | 2.7 | 0.4 | 12 | 8.83 | 0.69 | 0.2 | 0.04 | 8.52 | 0.63 | 9 |
| ***sIns*** | STEAM | 3 | 0.5 | 0.1 | 14 | 18.57 | 0.27 | 0.05 | 0.01 | 15.54 | 0.40 | 10 |
|  | STEAM | 7 | 0.2 | 0.1 | 26 | 16.84 | -0.31 | 0.03 | 0.01 | 17.63 | -0.25 | 9 |
|  | sLASER | 3 | 0.6 | 0.1 | 13 | 10.61 | 0.41 | 0.05 | 0.01 | 9.52 | 0.60 | 9 |
|  | sLASER | 7 | 0.2 | 0.1 | 16 | 14.31 | 0.86 | 0.03 | 0.01 | 13.41 | 0.86 | 10 |
| ***Tau*** | STEAM | 7 | 2.3 | 0.2 | 8 | 2.92 | 0.15 | 0.2 | 0.03 | 3.88 | 0.29 | 10 |
|  | sLASER | 7 | 2.1 | 0.3 | 9 | 7.56 | 0.70 | 0.2 | 0.03 | 9.75 | 0.63 | 9 |

*Table S4: Precentral gyrus metabolite concentration estimate, ratios to tCr, reliability and reproducibility measures at 3T without using the ppm gap*

| ***Metabolite*** | ***Sequence*** | ***Field Strength*** | ***Mean concentration estimate (std dev.)*** | | ***Mean CRLB (%)*** | ***Mean CV concentration estimate (%)*** | ***ICC using concentration estimate*** | ***Mean /tCr (std dev.)*** | | ***Mean CV tCr (%)*** | ***ICC tCr*** | ***N*** |
| --- | --- | --- | --- | --- | --- | --- | --- | --- | --- | --- | --- | --- |
| ***tCr*** | STEAM | 3 | 10.8 | 0.2 | 2 | 3.4 | 0.67 | 1.0 | 0.00 | 0.00 | NA | 10 |
|  | sLASER | 3 | 10.9 | 1.2 | 5 | 5.9 | 0.30 | 1.0 | 0.00 | 0.00 | NA | 10 |
| ***GABA*** | STEAM | 3 | 0.6 | 0.8 | 356 | 118.1 | 0.09 | 0.06 | 0.05 | 118 | 0.08 | 3 |
| ***Gln*** | STEAM | 3 | 2.07 | 0.5 | 19 | 19.1 | 0.72 | 0.1 | 0.05 | 19.2 | 0.72 | 10 |
| ***Glu*** | STEAM | 3 | 10.6 | 0.5 | 4 | 5.6 | 0.23 | 0.9 | 0.05 | 5.9 | 0.53 | 10 |
|  | sLASER | 3 | 10.7 | 2.7 | 25 | 7.2 | -0.71 | 1 | 0.2 | 4.4 | -0.71 | 10 |
| ***Glx*** | STEAM | 3 | 12.6 | 0.8 | 3 | 7.2 | 0.72 | 1.1 | 1 | 6.3 | 0.87 | 10 |
|  | sLASER | 3 | 19.7 | 4.8 | 11 | 8.4 | 0.61 | 1.8 | 0.3 | 5.6 | 0.09 | 10 |
| ***Lac*** | STEAM | 3 | 2.7 | 2.1 | 25 | 31.9 | 0.38 | 0.2 | 0.2 | 41.58 | 0.48 | 7 |
| ***tNAA*** | STEAM | 3 | 16.7 | 0.8 | 1 | 4.6 | 0.31 | 1.5 | 0.08 | 5.8 | -0.34 | 10 |
|  | sLASER | 3 | 22 | 3 | 3 | 4.2 | 0.45 | 2 | 0.1 | 4.3 | 0.53 | 10 |

| ***Metabolite*** | ***Sequence*** | ***Field Strength*** | ***Mean concentration estimate (std dev.)*** | | ***Mean CRLB (%)*** | ***Mean CV concentration estimate (%)*** | ***ICC using concentration estimate*** | ***Mean /tCr (std dev.)*** | | ***Mean CV tCr (%)*** | ***ICC tCr*** | ***N*** |
| --- | --- | --- | --- | --- | --- | --- | --- | --- | --- | --- | --- | --- |
| ***tCr*** | STEAM | 3 | 10.8 | 0.7 | 2 | 3.4 | 0.76 | 1.0 | 0.00 | 0.00 | NA | 10 |
|  | sLASER | 3 | 11.3 | 0.8 | 2 | 5.9 | 0.28 | 1.0 | 0.00 | 0.00 | NA | 10 |
| ***GABA*** | STEAM | 3 | 0.4 | 0.5 | 294 | 118.1 | -0.54 | 0.1 | 0.07 | 118 | -0.54 | 3 |
|  | sLASER | 3 | 1.6 | 1.4 | 277 | 90.6 | 0.58 | 0.1 | 0.2 | 93 | 0.55 | 9 |
| ***Gln*** | STEAM | 3 | 2.3 | 0.6 | 15 | 19.1 | 0.47 | 0.2 | 0.09 | 19.2 | 0.54 | 10 |
|  | sLASER | 3 | 2.5 | 1.4 | 44 | 29.3 | 0.71 | 0.2 | 0.1 | 30 | 0.72 | 10 |
| ***Glu*** | STEAM | 3 | 11.0 | 0.9 | 4 | 5.6 | 0.19 | 1.0 | 0.09 | 5.9 | 0.52 | 10 |
|  | sLASER | 3 | 12.5 | 1.3 | 7 | 7.2 | 0.06 | 1.9 | 0.08 | 4.4 | 0.58 | 10 |
| ***Glx*** | STEAM | 3 | 13.3 | 2 | 4 | 7.2 | 0.37 | 1.3 | 0.1 | 6.3 | 0.57 | 10 |
|  | sLASER | 3 | 15 | 2.3 | 6 | 8.4 | 0.46 | 1.3 | 0.04 | 5.6 | 0.75 | 10 |
| ***Lac*** | STEAM | 3 | 2.2 | 2.6 | 17 | 39.7 | 0.34 | 0.2 | 0.2 | 39.58 | 0.43 | 7 |
|  | sLASER | 3 | 3.3 | 3.6 | 22 | 27.67 | 0.45 | 0.2 | 0.3 | 67.63 | 0.57 | 9 |
| ***tNAA*** | STEAM | 3 | 15.9 | 1 | 1 | 4.6 | 0.19 | 1.5 | 0.1 | 5.8 | 0.38 | 10 |
|  | sLASER | 3 | 17.6 | 1.2 | 2 | 4.2 | 0.63 | 1.5 | 0.08 | 4.3 | 0.34 | 10 |

*Table S5: Paracentral lobule metabolite concentration estimate, ratios to tCr, reliability and reproducibility measures at 3T without using the ppm gap*

**12.1. Phantom experiments**

The mean ± standard deviation of tCr linewidth and tCr SNR were 5.2 ± 0.71 Hz and 2976 ± 44 for sLASER at 7T, 4.4 ± 0.6 Hz and 1260 ± 71 for STEAM at 7T, 3.1 ± 0.52 Hz and 1673 ± 69 for sLASER at 3T, and 2.8 ± 0.12 Hz and 782 ± 65 for STEAM at 3T, respectively. The following metabolites were quantified over five sessions: Cr, GABA, Glu, Ins, Lac, tCho, and NAA. Mean ± standard deviation for all metabolite ratios to tCr, along with reproducibility assessments, are summarized in Supplementary Table S4.

**12.1.1. sLASER vs STEAM**

The results indicated that tCr linewidths between sequences is not statistically significant (p = 0.25), while tCr SNR was significantly higher for sLASER at the same field strength (p = 0.0001). Notably, a paired t-test between the two sequences revealed a significantly lower NAA CRLBs for sLASER compared to STEAM (p < 0.001). The mean CRLB differences for all other metabolites were not statistically significant (p > 0.05). CVs for most metabolites were lower for sLASER than STEAM at both field strengths, however, the differences were not statistically significant (p > 0.1).

**12.1.2. 3T vs. 7T**

As expected, SNR and linewidth (in Hz) increased with the field strength due to the heightened chemical shift dispersion and SNR, aligning with the findings of other studies ^1^. CRLBs for all metabolites remained consistently low, with values below 15% for both sLASER and STEAM sequences at 7T and below 25% at 3T. The mean CRLB values between field strengths for Glu, Ins, Lac, and tCho were not statistically significant (p > 0.05) except for GABA CRLB values at 7T compared to 3T (p = 0.03). In the comparison between field strengths, GABA, tCho, Lac, and NAA consistently exhibited a trend of lower CVs at 3T compared to 7T for both sequences (p > 0.1).

Table S6. Metabolite ratios to tCr and reproducibility measures from phantom scans.

| ***Sequence*** | ***Field strength*** | ***Metabolites*** | ***Mean CV (%)*** | ***Mean ratio to tCr (std dev.)*** | ***Mean CRLBs*** |
| --- | --- | --- | --- | --- | --- |
| STEAM | 3 | Cho | 1.8 | 0.3 (0.01) | 4.0 |
|  |  | GABA | 8.0 | 0.4 (0.03) | 24.4 |
|  |  | Glu | 2.4 | 1.4 (0.04) | 5.6 |
|  |  | Ins | 0.6 | 1.0 (0.01) | 4.6 |
|  |  | Lac | 1.0 | 0.6 (0.01) | 7.4 |
|  |  | NAA | 1.0 | 1.3 (0.01) | 3.0 |
| STEAM | 7 | Cho | 2.9 | 0.3 (0.01) | 4.4 |
|  |  | GABA | 15.1 | 0.2 (0.04) | 15.0 |
|  |  | Glu | 1.0 | 1.5 (0.02) | 4.8 |
|  |  | Ins | 1.0 | 0.8 (0.01) | 5.8 |
|  |  | Lac | 4.8 | 0.5 (0.03) | 8.0 |
|  |  | NAA | 1.4 | 1.5 (0.02) | 3.0 |
| sLASER | 3 | Cho | 0.1 | 0.3 (0.00) | 3.3 |
|  |  | GABA | 2.3 | 0.4 (0.01) | 21.6 |
|  |  | Glu | 0.8 | 1.6 (0.01) | 4.4 |
|  |  | Ins | 2.1 | 0.9 (0.02) | 5.4 |
|  |  | Lac | 2.0 | 0.6 (0.01) | 9.2 |
|  |  | NAA | 0.7 | 1.6 (0.01) | 2.0 |
| sLASER | 7 | Cho | 0.6 | 0.3 (0.00) | 3.0 |
|  |  | GABA | 2.6 | 0.3 (0.01) | 11.0 |
|  |  | Glu | 0.7 | 1.4 (0.01) | 3.2 |
|  |  | Ins | 0.8 | 0.9 (0.01) | 3.0 |
|  |  | Lac | 3.3 | 0.6 (0.02) | 4.8 |
|  |  | NAA | 0.8 | 1.3 (0.01) | 2.0 |

Table S7. THE MRSinMRS CHECKLIST

| 1. Hardware | | | | |
| --- | --- | --- | --- | --- |
| a. Field strength (T) | 3 T | | 7 T | |
| b. Manufacturer | Siemens | | Siemens | |
| c. Model | ve syngo MR E11 | | vd syngo MR E12 | |
| d. RF coils | 62 ch ^1^H head coil | | 32 ch ^1^H head coil | |
| 2. Acquisition | | | | |
| a. Pulse sequence | STEAM | sLASER | STEAM | sLASER |
| b. VOI locations | Precentral Gyrus and Paracentral Lobule | | | |
| c. VOI size (mm^3^) | 25x25x25 mm^3^ | | | |
| d. TR/TE (ms) | 2000/10 | 2000/28 | 8000/8 | 8000/26 |
| e. Averages | 64 | | 16 | |
| f. TM, spectral width in Hz, number of spectral points | 43, 2500, 2048 | N/A, 4000, 2048 | 32, 6000, 2048 | N/A, 6000, 2048 |
| g. Water suppression method | VAPOR | | | |
| h. Shimming method | fastestmap | | | |
| 3. Data analysis methods and outputs | | | | |
| a. Analysis software | Osprey | | | |
| b. Processing steps deviating from quoted reference or product | Default Osprey | | | |
| c. Output measure | Ratio/tCr and metabolite concentration estimate (Tissue and relaxation corrected) | | | |
| d. Quantification references and assumptions, fitting model assumptions | Basis set includes 19 simulated metabolites + measured macromolecule. LCModel basline knot spacing 5.00 ppm | | | |
| 4. Data quality | | | | |
| a. Reported variables (SNR, linewidth (with reference peaks)) | SNR dividing the tCr by the standard deviation of noise within the range of -2 to 0 ppm. Linewidth FWHM of a Lorentzian peak model for the water peak between 4.4 and 5.0 ppm. (Figure 3) | | | |
| b. Data exclusion criteria | CRLB > 50% | | | |
| c. Quality measures of postprocessing model fitting (eg CRLB, goodness of fit, SD of residual) | CRLB, and SD of residual | | | |
| d. Sample spectrum | Figure 2 | | | |


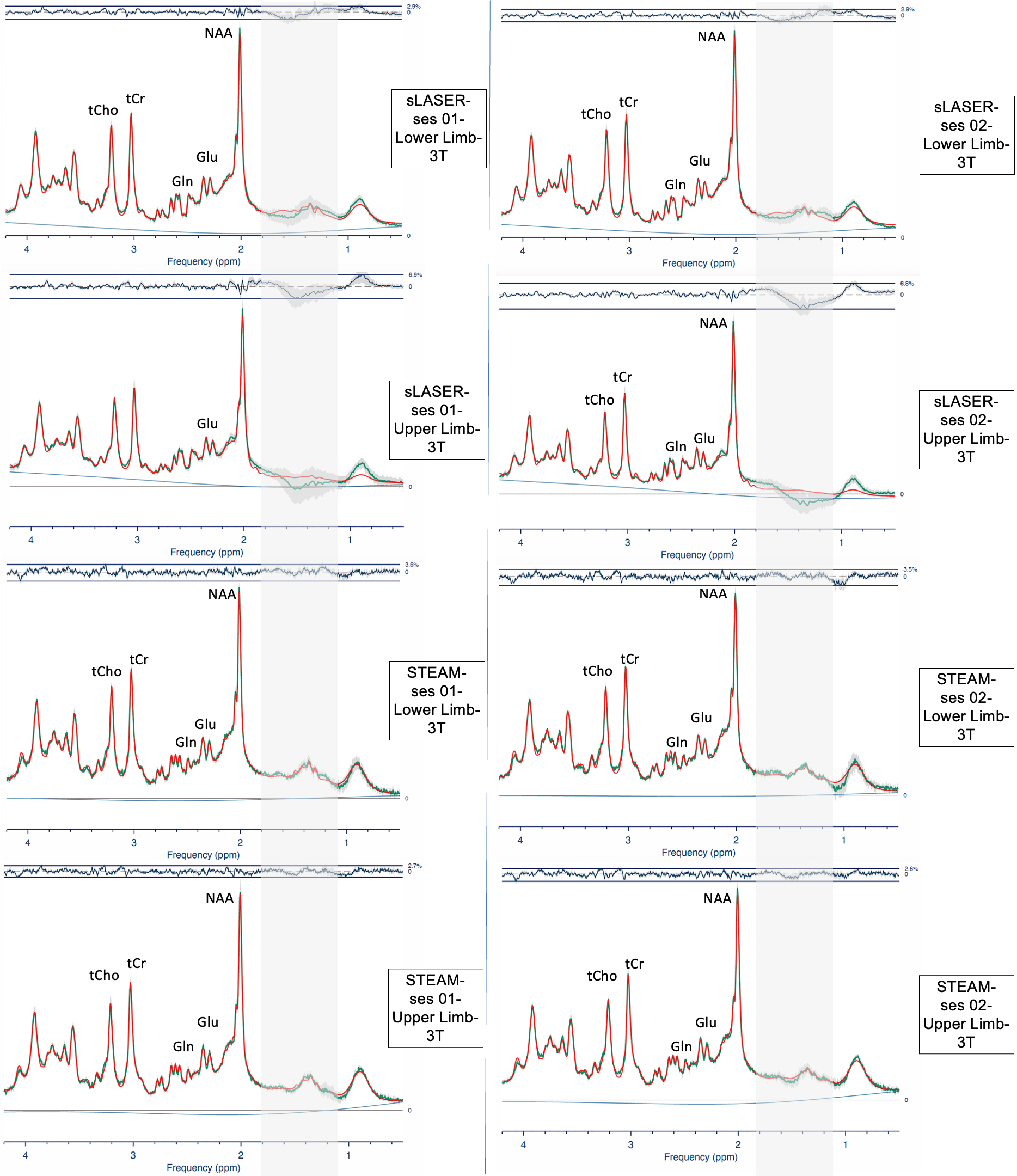


*Figure S1: Average (full line) and standard deviation (shaded area) of individual spectra are shown for 5 volunteers at 3 T for two locations (upper limb and lower limb) from session 1 (left side) and session 2 (right side) for both sequences (sLASER and STEAM). A gap from 1.1 to 1.85 ppm was used to account for potential lipid contamination and represented a region of lower fitting confidence.*


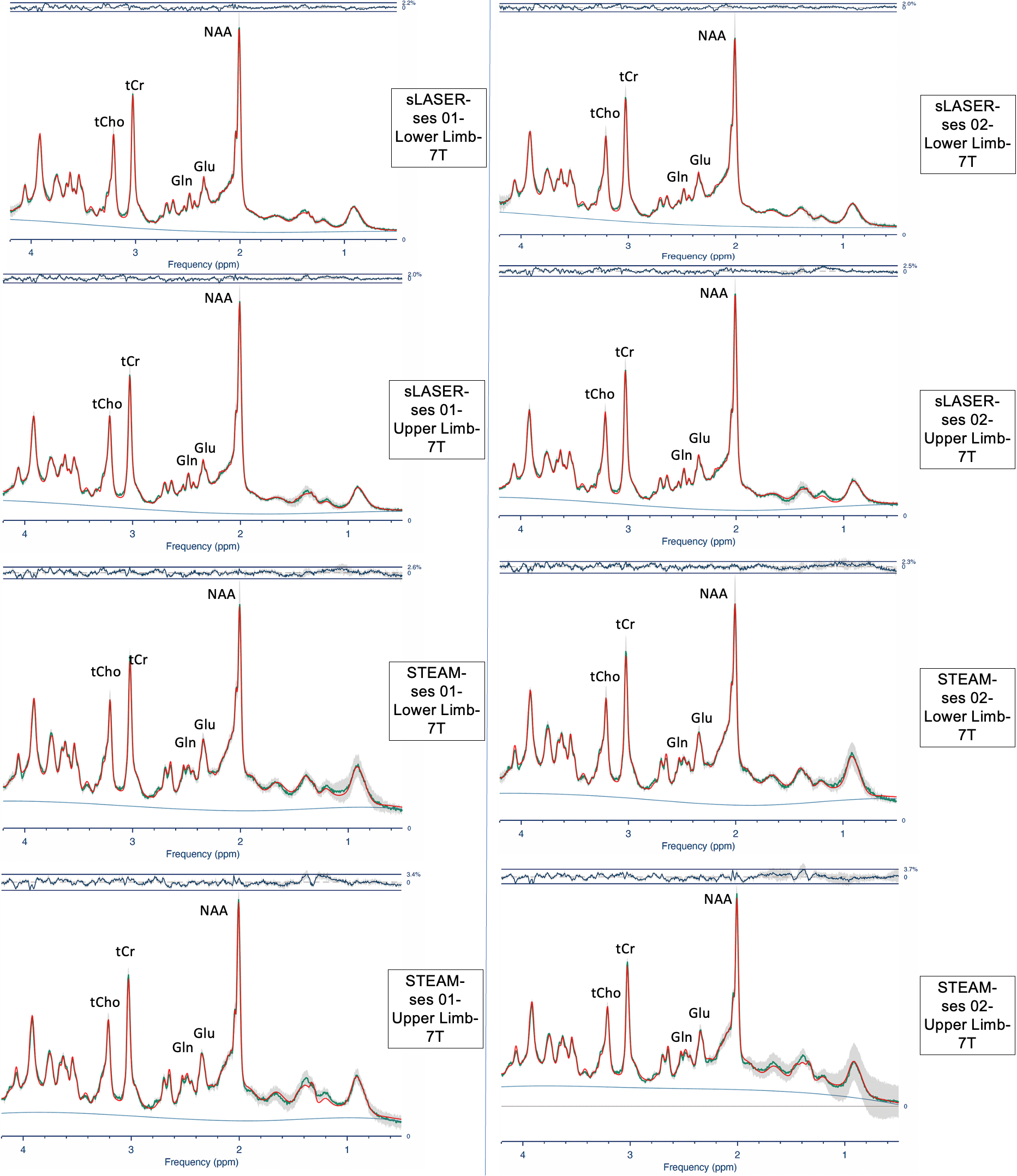


*Figure S2: Average (full line) and standard deviation (shaded area) of individual spectra are shown for 5 volunteers at 7 T for two locations (upper limb and lower limb) from session 1 (left side) and session 2 (right side) for both sequences (sLASER and STEAM).*

1. Pradhan S, Bonekamp S, Gillen JS, et al. Comparison of single voxel brain MRS AT 3T and 7T using 32-channel head coils. *Magnetic Resonance Imaging*. Oct 2015;33(8):1013-8. doi:10.1016/j.mri.2015.06.003
